# 302. Using Antiphospholipid Antibody Presence as an Additional Biomarker to Identify COVID-19 Positive Patients with High Risk for Thrombosis

**DOI:** 10.1093/ofid/ofab466.504

**Published:** 2021-12-04

**Authors:** Jennifer R Hewlett, Jing Du, M Sung Lee, Gavin McLeod, Herbert Archer

**Affiliations:** Greenwich Hospital, Greenwich, Connecticut

## Abstract

**Background:**

Patients who are hospitalized with Coronavirus 2019 (COVID-19) are known to have increased risk for thrombosis. Several mechanisms have been proposed for increased thrombogenesis, including antiphospholipid antibodies (APLs). We sought to better understand the relationship between a commonly used marker of thrombosis, D-dimer, and antiphospholipid antibodies in relation to thrombosis in COVID-19.

**Methods:**

This was a single-center prospective cohort study. Participants were adults admitted to the hospital with COVID-19 between March and December of 2020. Included patients required a positive COVID-19 nasopharyngeal nucleic acid amplification testing (NAAT), coagulation studies, and regular assessment of D-dimer levels. Patients who were excluded were pregnant adults, use of oral anticoagulants prior to admission, and absence of a positive COVID-19 nasopharyngeal NAAT. We tested 52 patients for antiphospholipid antibodies (APLs), including lupus anticoagulant (LA), anti-beta-2 glycoprotein antibodies (B2GP), and anti-cardiolipin antibodies (aCL). The endpoint for analysis was hospital discharge or development of a confirmed thrombosis.

**Results:**

Twenty-nine of fifty-two patients (55.7%) with COVID-19 had non-negative APLs. Of these patients, twenty-seven (93.1%) had non-negative aCLs, the majority of which were IgM antibodies. There was a total of 7 thrombotic events in our cohort. The sensitivity of D-dimer alone was 85% and the sensitivity of APLs alone was 71%. In patients with an intermediate D-dimer level (i.e., greater than 2 milligrams per liter (mg/L) but less than 5 mg/L), the addition of non-negative APLs increased the sensitivity of D-dimer to 100%. In patients with a high D-dimer (i.e., greater than 5), the combined sensitivity of D-dimer and APLs was 60%. Out of the 7 thrombotic events in our cohort, two patients had negative APLs, however both patients had a D-dimer of greater than 5 mg/L.

**Conclusion:**

The use of APLs can assist in risk-stratifying patients in an intermediate-risk D-dimer group to consider prophylactic anticoagulation if APLs are negative and to consider therapeutic anticoagulation if APLs are non-negative. In the high-risk group (i.e., a D-dimer greater than 5 mg/dL), a therapeutic anticoagulation approach may be more appropriate.

**Disclosures:**

**All Authors**: No reported disclosures

